# Metal Organic Frameworks Derived Sustainable Polyvinyl Alcohol/Starch Nanocomposite Films as Robust Materials for Packaging Applications

**DOI:** 10.3390/polym13142307

**Published:** 2021-07-14

**Authors:** Naveed Ahmed Khan, Muhammad Bilal Khan Niazi, Farooq Sher, Zaib Jahan, Tayyaba Noor, Ofaira Azhar, Tazien Rashid, Naseem Iqbal

**Affiliations:** 1 Department of Chemical Engineering, School of Chemical and Materials Engineering, National University of Sciences and Technology, Islamabad 44000, Pakistan; naveedahmed@uspcase.nust.edu.pk (N.A.K.); m.b.k.niazi@scme.nust.edu.pk (M.B.K.N.); zaibjahan@scme.nust.edu.pk (Z.J.); tayyaba.noor@scme.nust.edu.pk (T.N.); ofairaazhar@gmail.com (O.A.); 2 Department of Engineering, School of Science and Technology, Nottingham Trent University, Nottingham NG11 8NS, UK; 3 Department of Chemical Engineering, NFC Institute of Engineering and Fertilizer Research, Faisalabad 38000, Pakistan; tazien@iefr.edu.pk; 4 U.S.-Pakistan Center for Advanced Studies in Energy (USPCAS-E), National University of Science and Technology, Sector H-12, Islamabad 44000, Pakistan; naseem@casen.nust.edu.pk

**Keywords:** sustainable polymers, MOFs, ZIF-67, solvothermal synthesis, PVA/starch, bio-nanocomposite films

## Abstract

Bio-nanocomposites-based packaging materials have gained significance due to their prospective application in rising areas of packaged food. This research aims to fabricate biodegradable packaging films based upon polyvinyl alcohol (PVA) and starch integrated with metal-organic frameworks (MOFs) or organic additives. MOFs offer unique features in terms of surface area, mechanical strength, and chemical stability, which make them favourable for supporting materials used in fabricating polymer-based packaging materials. zeolitic imidazolate frameworks (ZIFs) are one of the potential candidates for this application due to their highly conductive network with a large surface area and high porosity. Present research illustrates a model system based on ZIF-67 (C_8_H_10_N_4_Co) bearing 2–10 wt.% loading in a matrix of PVA/starch blend with or without pyrolysis to probe the function of intermolecular interaction in molecular packing, tensile properties, and glass transition process. ZIF-67 nanoparticles were doped in a PVA/starch mixture, and films were fabricated using the solution casting method. It was discovered through scanning electron microscopy (SEM), X-ray diffraction (XRD), thermogravimetric analysis (TGA), and Fourier transform infrared spectroscopy (FTIR) that addition of ZIF-67 and pyrolyzed ZIF-67 changed and enhanced the thermal stability of the membrane. Moreover, 2–10 wt.% loading of ZIF-67 effected the thermal stability, owing to an interlayer aggregation of ZIF-67. The membranes containing pyrolyzed ZIF-67 showed mechanical strength in the order of 25 MPa in a moderate loading of pyrolyzed ZIF-67 (i.e., at 4 wt.%). The crystallinity enhanced by an increment in ZIF-67 loading. On the other hand, pyrolyzed ZIF-67 carbon became amorphous because of the inert environment and elevated temperature. The surface area also increased after the pyrolysis, which helped to increase the strength of the composite films.

## 1. Introduction

Over the years, the use of packaging has gained much attention due to the increase in international trade and the rise in global warming. Biodegradable packaging materials are replacing petroleum-based non-biodegradable materials. The use of polyvinyl chloride (PVC), polyethylene terephthalate (PET), and polystyrene materials results in the pollution of the oceans as well as an increase in greenhouse gases during manufacturing and decomposing processes, consequentially leading to elevated global average temperatures [1]. Good mechanical and thermal properties as well as the low cost of petro-based polymers enhance their use, but they are the main reason for global warming. If the focus is only on sustainability and not on the reliability, the quality and functionality of a product is bound to fail in the cutthroat economic conditions of the present day. The development of biodegradable materials is one of the solutions in diminishing human impact on the environment [2]. Environmentally friendly business policies require environmental benign packaging materials that can bear the harsh conditions for packaging applications and can degrade easily without polluting the environment [3].

Polymer composites have been widely considered as a replacement for electromagnetic interference (EMI) shielding materials because of their low density, processing capability, and stability [4]. For packaging materials, the blending of plastics and complex sugars such as carboxymethyl cellulose (CMC), starch, and cellophane can result in products that biodegrade at a faster rate. Starch has long been considered to be a biopolymer with immense potential for use in biodegradable plastics [5]. Intrinsic biodegradability, enormously abundant in nature, cheap, and the capability to be processed using traditional plastic processing equipment are the properties that make starch an auspicious natural polymer [6]. Starch lacks processability with common industrial equipment such as extruders and thermoforming presses, poor mechanical properties after being thermoformed (brittleness), high density (1400 kg/m^3^), and finally, it is hydrophilic. Therefore, starch on its own cannot be used as a packaging material. However, this challenge can be easily addressed by mixing it with plastics such as polycaprolactone (PCL), polylactic acid (PLA), PVA, etc. [7].

polyvinyl alcohol (PVA) is a nontoxic biocompatible and biodegradable polymer in nature [8]. It is an ivory odourless and tasteless powder that can be easily physically or chemically mixed with other polymers. PVA is considered an environmental friendly food packaging material that shows excellent water absorption, is chemical and gas resistant, and has film-forming properties [9]. The incorporation of nanoparticles in PVA/starch blend films has enhanced the mechanical, thermal, and electrochemical properties remarkably [10]. Nano-fillers such as clay, organic, inorganic, or carbon nanostructures have also been recognized to prompt higher tensile strength and ameliorate hurdles against gasses and water vapour [11]. These additives can improve barrier and mechanical properties by decreasing filler proportions and reducing production costs due to less material consumption [12]. The mechanical features of the nanocomposites mainly influence the thickness, modulus, and strength of the polymer matrix [13]. To enhance starch film properties like mechanical properties, water absorption behaviour, and in vitro degradation of the films, nano-cellulose is added to the starch. Depending on the results, nano-cellulose leads to the improvement of the mechanical properties of starch by up to 70% [14].

Nanocrystalline cellulose has been used in the starch chitosan and gelatin-chitosan nanocomposite films as a nanoparticle additive. To enhance antibacterial and antifungal properties, chitosan has been utilized as an additive. The potential of natural fibres as a reinforcement for composites has been analyzed in various studies [15]. PVA and starch-based packaging film is also reported in the literature, and it was observed that the molecular weight of PVA does not affect water vapour permeability but does strongly affect tensile strength [16]. PVA can be homogeneously mixed with starch to make new packaging materials, but the combination of PVA and starch decreases the mechanical thermal properties and also lowers the resistance to moisture in comparison to pure PVA [17]. Moreover, this can be improved by introducing metal-organic frameworks (MOFs) into the process to incorporate stresses into the system, thereby improving strength [18]. MOFs are also considered porous multifunctional polymers. They are synthesized by the mixing of metal ions (Zn^2+,^ Cu^2+^, Co^2+^ Cr^3+^, Al^3+^, Fe^3+^ and Zr^4+^) and an organic linker (carboxylates, or anions, such as sulfonate, phosphonate, heterocyclic compounds, and 2-methylimidazole).

MOFs can be characterized based on the metal ions used, the adopted synthesis procedure, and the crystal structure of the resultant material [19]. MOFs can also be used in conjunction with other materials (functional components/matrix) to form composites with extraordinary applications such as drug delivery/release, fuel cell, and electrochemical catalysis [20]. MOFs can be built up by using two main components known as connectors and linkers. Connectors appear as transition metal ions with different oxidation states, while linkers give wide opportunities for linking sites that provide binding strength and directionality [21]. The MOF subcategory ZIF-67 is an attractive form due to its high porosity, synthesis capability, high surface area, solubility, and solvent capability compared to other MOFs. ZIF-67 is promising for use in adsorption technology and is mainly synthesized from cobalt ion and 2-methylimidazolate. Different combinations of imidazole linkers and metal ions determine the structure, properties, and type of the ZIFs. Recently discovered zinc-based imidazole frameworks have shown porous structures that are symmetrical and having a similar structural analogy to zeolites. The most notable examples of these include ZIF-67 and ZIF-8 [22].

The key objective of this research is to investigate the impact of ZIF-67 stacking on the morphology of PVA/starch films. The glass transition temperature (Tg) of the cross-connected PVA layer increases in specific and deliberate increments with loading the zeolite, showing that the vacant spaces of cross-connected PVA film diminish by expanding the zeolite stacking. This is due to the fact that the segmental movements of the chains are limited by expanding the zeolite stacking. Normally, an expansion in free volume prompts higher permeation flux, which minimizes the selectivity [23]. Keeping this background, a study was conducted to fabricate and characterize ZIF-67 based PVA/Starch nanocomposite films for packaging applications. Both ZIF-67 and pyrolyzed ZIF-67 incorporated with PVA/starch films were characterized and compared in terms of their thermo-mechanical properties. Previously, ZIF-8 has been investigated by Sharma et al. [24]. Here, we have synthesized and analyzed ZIF-67 with and without pyrolysis, and contrary to using ethanol in the synthesis, we have considered the deionized water pathway. Fourier transform infrared spectroscopy (FTIR) and scanning electron microscopy (SEM) were performed to study the interaction of functional groups and the morphology of films, respectively. Phase identification and X-ray diffraction (XRD) were used to investigate the crystallinity of the films.

## 2. Experimental

### 2.1. Materials and Methods

Cobalt Nitrate Hexahydrate (Co (NO_3_)_3_·6H_2_O) was of 97% purity and was procured from DAEJUNG Chemical (DAEJUNG CHEMICAL & METALS CO., LTD., Gyeonggi-Do, Korea). The 2-methylimidazole of 99% purity, polyvinyl alcohol (PVA; MW 1500), and starch were acquired from Merck, NY, USA. All of these chemicals were used as received without any further purification.

### 2.2. MOF’s Synthesis

ZIF-67 (C_8_H_10_N_4_Co) was synthesized by creating a solution with 1.97 g of 2-methylimidazole in 20 mL of deionized water. Simultaneously, 1.74 g of cobalt nitrate was mixed in 20 mL of de-ionized water as well. The stirring of both the solutions was done for 20 h at 25 °C. The purple precipitates formed, which were then centrifuged followed by being washed 2–3 times with water. The collected precipitates were dried at 80 °C overnight. To compare the effect of pyrolysis on the strength of the final film, the prepared ZIF-67 was also pyrolyzed in a tube furnace at an ultimate temperature of 700 °C under an argon atmosphere. The structure of the ZIF-67 nanoparticles depends on the calcination temperature [25]. The pyrolysis process was conducted by heating from 50 to 350 °C at a ramp rate of 10 °C/min and then to 700 °C at a rising rate of 2 °C/min. The temperature was held at 350 °C for 1.5 h and then at 700 °C for 2 h. The resultant product contained less than half of the mass of the initial weight of the sample [26]. All experiments were performed in a set of three replicates.

### 2.3. Preparation of PVA-Starch-ZIF-67 Films

PVA 5 g was added to 50 mL of distilled water followed by the addition of 3.5 g of starch in 50 mL water in two separate beakers. The solutions were stirred at 80 °C for 2 h and were subsequently mixed and stirred for another 2 h at the same temperature. A total 5–6 drops of glutaraldehyde for cross-linking and glycerin as a plasticizer were also added to the film at this stage [27]. Finally, the ZIF-67 was added to the resultant mixture with different wt.% according to the amount of PVA as shown in Table 1. A series of composites were prepared by mixing 2 to 10 wt.% ZIF-67 in a PVA/starch blend. All of the prepared compositions were sonicated for an extra 30 min. to achieve homogenization. A detailed description of the amounts of additives (ZIF-67) is given in Table 1. A similar synthesis procedure was repeated for the pyrolyzed ZIF-67 as well in order to create six additional films. The PVA/starch along with the ZIF-67 polymeric films were synthesized in Petri dishes with a dia. of 14 cm. The films were dried in a Petri dish for 48 h at 25 °C and desiccated in an oven at 50 °C for 5 h to ensure maximum drying.

## 3. Characterization

Characterization of the produced ZIF-67 was done using various techniques [28], i.e., scanning electron microscopy (SEM), ultimate tensile testing, X-Ray diffraction (XRD), Brunauer–Emmett–Teller (BET), thermogravimetric analysis (TGA), and Fourier transform infrared spectroscopy (FTIR). The above-mentioned techniques are discussed below.

### 3.1. SEM and Energy Dispersive X-ray Spectroscopy

SEM is used to study the morphology of synthesized material and films. To evaluate the surface morphology of the composite films and for elemental analysis, SEM-EDS analyzer JSM-6490A JEOL (Tokyo, Japan) were used, respectively. The accelerating voltage of the electron beam (HV) 20 and 30 kV was used for pure ZIF-67, and 10 kV was used for the composite films. For EDS analysis, a 20 kV electron beam and a working distance (WD) of 15 mm was maintained. Secondary electrons (SE) were used for both SEM and EDS analysis. A thin layer of palladium gold was coated before the analysis of the composites for better conductivity to generate high-resolution images [29].

### 3.2. X-ray Diffraction (XRD) Studies

X-rays are used to diagnose the framework and placement of different atoms within the crystal structure of a crystalline material [30]. The X-ray Diffraction Machine D8 Advance by Bruker, Karlsruhe, Germany was used to analyze the composites films. The source of the X-ray was a copper tube with a wavelength of 1.548 °A and a 40 kV and 30 mA voltage and current, respectively. Composites were scanned between the 2θ range of 05 to 70, with an increment of 0.02 degrees and 0.1 s step time.

### 3.3. Thermogravimetric Analysis (TGA)

The thermal gravimetric analysis and differential thermal analysis (DTA) involves the monitoring of the weight loss of a sample while it is being heated in order to gather information about the thermal stability of the material along with the processes of absorption, desorption, and phase transformations of the material [31]. The analysis was carried out simultaneously with the TG/DTA (DT-60) Shimadzu by Japan with a 10 °C per minute ramp rate from 25 to 600 °C in a N_2_ (200 mL/min) atmosphere.

### 3.4. Ultimate Tensile Testing

A universal tensile machine (UTM) AGS-X by Shimadzu, Kyoto, Japan was used to investigate the mechanical strength of the composite films. A crosshead speed of 10 mm/min for the test was conducted. The 0.63 mm ± 0.05 mm thickness and the 50 mm gauge length of the samples were measured [32]. This test was repeated three times for all of the samples to confirm the repeatability of the results.

### 3.5. Fourier Transform Infrared (FTIR) Spectroscopy

The Fourier transform infrared spectroscopy was performed to investigate the interfacial interaction of the functional groups present in the material and films [33]. A source of light falls onto the sample, and radiations of the IR region are absorbed to give information about the functional groups. A Cary 630 (Agilent technologies, Santa Clara, CA, USA) FTIR spectrometer was used to obtain the FTIR spectrums of the PVA–starch composite films. Zinc selenide was used as a detector along with a diffused reflectance accessory for testing the samples over a wavelength range of 4000 to 400 cm^−1^.

### 3.6. Brunauer-Emmett Teller (BET) Analysis

The Brunauer–Emmett–Teller (BET) analysis was performed by NOVA Quantachrome Instruments, Boynton Beach, FL, USA. The BET theory describes the phenomenon of the physical adsorption of gas on the surface of a material. This technique is used for the critical analysis of specific areas of materials. ZIF-67 has a zeolitic framework that possesses a large porosity that can be further reduced to cobalt nanoparticles and can catalyze the graphitization of obtained carbons afterwards. The surface area of ZIF-67 is increased due to the graphitization of carbon particles. The surface area is further increased when the pyrolysis temperature reaches between 800 and 900 °C [34]. The BET isotherm is evaluated by the monolayer formation of gas molecules adsorbed on the adsorbent surface.

## 4. Results and Discussion

### 4.1. Surface Morphological Analysis

**Figure 1:**
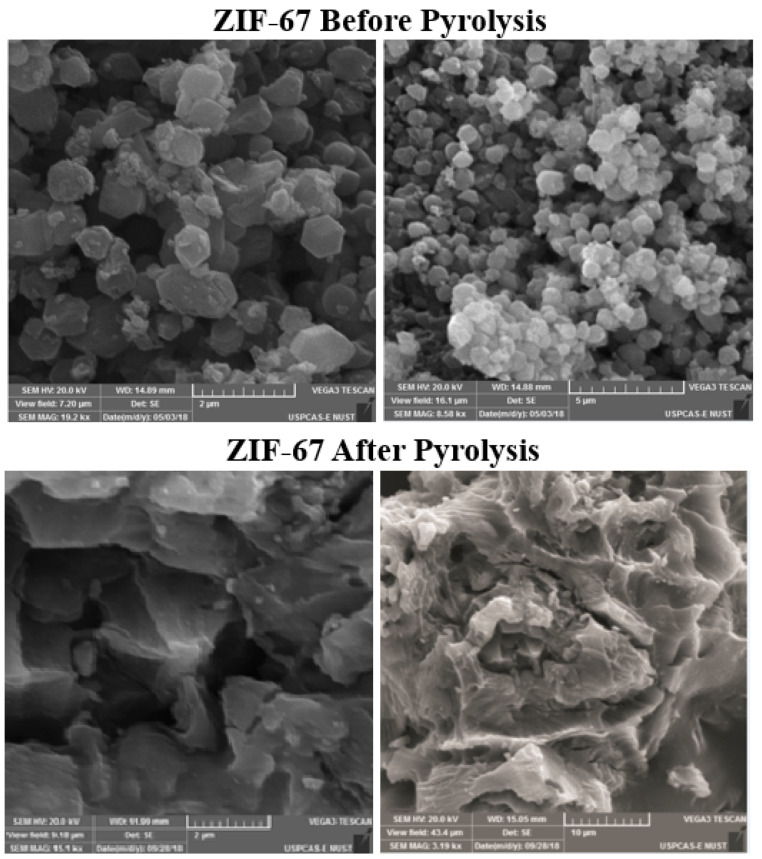
Surface morphology of MOFs ZIF-67 before and after pyrolysis.

**Figure 2:**
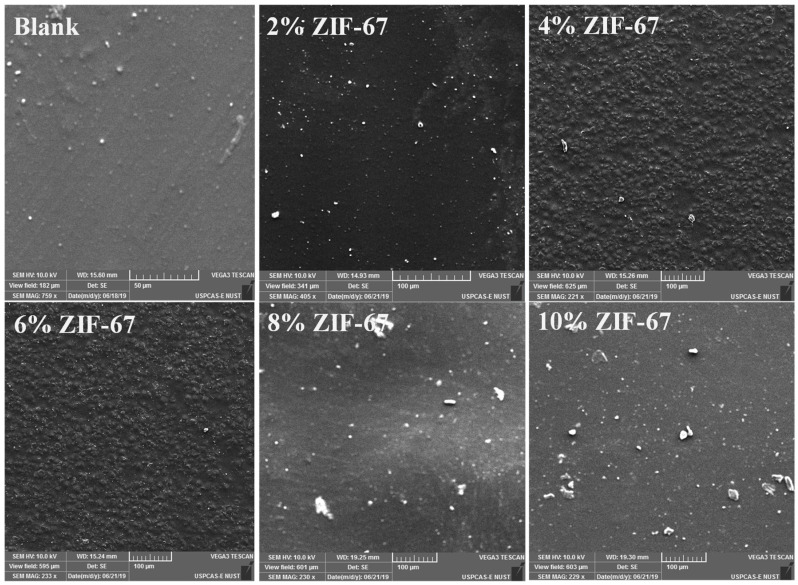
Surface morphology of PVA/starch nanocomposite films with various compositions of ZIF-67 SEM images: Blank, 2 wt.% ZIF-67, 4 wt.% ZIF-67, 6 wt.% ZIF-67, 8 wt.% ZIF-67, and 10 wt.% ZIF-67.

The nanostructure, dispersion, and scattering of ZIF-67 in the PVA/starch film were observed by SEM. Figure 1 represents the morphology of both surfaces: ZIF-67 and pyrolyzed ZIF-67. It showed partial agglomeration, as the concentration of the ZIF-67 increased, which may show low compatibility with the polymer matrix and reduce selectivity [35]. The surface area of the MOFs increases as the ZIF-67 is pyrolyzed. Figure 2 shows the surface morphology of the nanocomposite films blends with non-pyrolyzed ZIF-67. The homogeneous film without ZIF-67 has a smooth surface that showed the distribution and polymer compatibility. A gradual increase in the aggregation and the heterogeneity of the samples with the addition of the ZIF-67 are shown in Figure 1. Around 4 wt.% MOF can be distributed uniformly in the PVA starch composite films.

**Figure 3:**
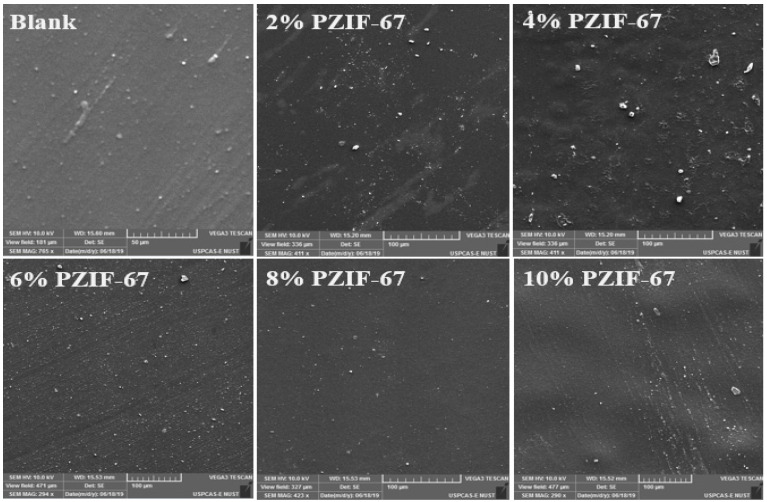
Surface morphology of PVA/starch homogeneous films with various compositions of pyrolyzed ZIF-67 SEM images: Blank, 2 wt.% PZIF-67, 4 wt.% PZIF-67, 6 wt.% PZIF-67, 8 wt.% PZIF-67, and 10 wt.% PZIF-67.

However, as the concentration is increased further, the added ZIF-67 seems to aggregate in Figure 2. Due to the agglomeration of the nanoparticles in the composite, the surfaces became porous and clustered with the increased concentration of the ZIF-67. This may be attributed to the affinity of ZIF-67 to form aggregates above 5 wt.% concentrations [34]. The pyrolyzed ZIF-67 texture properties vary from non-pyrolyzed ZIF-67. Figure 3 shows the morphology of the surface of the PVA/starch blend films with pyrolyzed ZIF-67. Pyrolyzed ZIF-67 shows a higher accumulation of nanoparticles on the surface in comparison to the non-pyrolyzed ZIF-67 due to the high porosity [36]. The surface area of the pyrolyzed ZIF-67 increases, which forms more homogenous blends with PVA/starch and distributes ZIF-67 more uniformly, up to 4 wt.% of the ZIF-67 sample. Even after this composition, the increase in the amount of pyrolyzed ZIF-67 in the composition showed less agglomeration and more dispersion compared to the pyrolyzed ZIF samples.

The maximum dispersion of ZIF-67 is in the 4 wt.% sample. The better dispersion improves the mechanical properties of the pyrolyzed ZIF-67 as shown in tensile testing results is because of greater interfacial interaction. The decrease in ductility indicates the enhancement of rigidity due to a greater extent of interfacial interaction between composite films and MOFs [37]. Mass channel transfer, strength, and stability of the film enhance due to these porous structures; therefore, enhancing the oxygen permeability property of the packaging material [38]. The amount of ZIF-67 dispersed in the composite films was determined using EDS [39]. Table 2 shows the results of the EDS analysis. The ZIF-67 showed uniform distribution up to 4 wt.%, but at 6 wt.% and above, ZIF-67 loading shows the agglomeration between the composite films and ZIF-67. After the pyrolysis, ZIF-67 becomes more porous with a large surface area, which increases the interfacial interaction between the composites films and the pyrolyzed ZIF-67 [40]. The SEM results justified this by showing a more uniform distribution and less agglomeration of the pyrolyzed ZIF-67 on the membrane surface than non-pyrolyzed ZIF-67 samples.

### 4.2. Crystallinity Analysis

**Figure 4:**
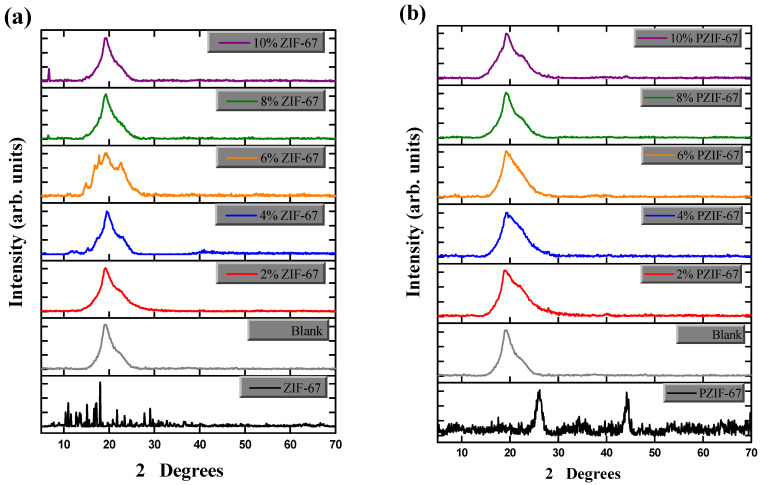
X-ray diffraction analysis of PVA/starch films. (**a**) Different compositions of ZIF-67 (0, 2, 6, 8, and 10%); (**b**) Various amount of Pyrolyzed ZIF-67 (0, 2, 4, 6, 8, and 10%).

The XRD patterns for the composite films and ZIF-67 were obtained using a D8 Advance Bruker, Karlsruhe, Germany X-ray diffractometer with powder Cu-Kα (1.58 Å) radiation. The diffraction patterns of ZIF-67 and the composite films are shown in Figure 4a,b. The diffraction peak of PVA can be seen in the blank spectrum with a peak of ~18.5° at 2θ degrees. These are typical crystallites of PVA that are overlapped with a wide hump showing the characteristic of an amorphous region [30]. The enhanced diffraction peaks mean that the crystallinity of the ZIF-67 MOFs has slightly improved and that effective results were obtained due to pyrolysis. Purity control and the microstructure of the resultant product is dependent on the heat treatment. [32]. The ZIF-67 crystallinity structure has formed a favourable crystal structure on the blend film, enhancing the mechanical support and structural stability.

Being a crystalline substance, ZIF-67 shows clear peaks in the diffractogram. PZIF-67 also shows a clear peak due to the crystalline structure. XRD peaks become sharper with the ZIF-67 and PZIF-67 composite films as the composition of ZIF-67 is enhanced. This shows that the crystallinity of ZIF-67 remains unchanged when it is added to PVA–starch films. However, in pyrolyzed ZIF-67, the carbon becomes amorphous, even at a higher temperature, and only the cobalt peaks showed the crystalline structure. An even further increase in pyrolysis temperature increased the cobalt nanoparticles crystallinity gradually [32]. Hexagonal shaped crystals can be seen by the SEM due, and it is these hexagonal shaped crystals that enhance the thermal stability and strength of the nanocomposite films.

### 4.3. Thermal Stability Analysis

**Figure 5:**
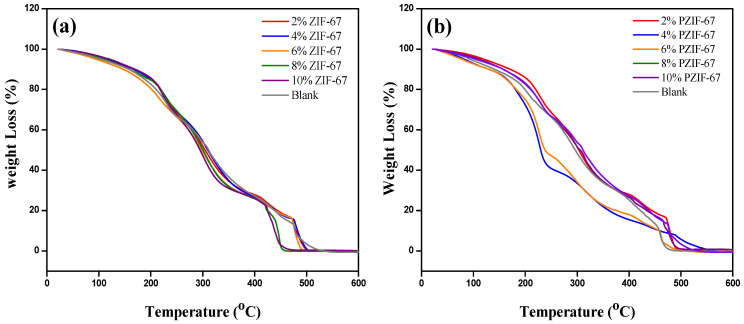
Weight loss analysis by TGA of PVA/starch nanocomposite films with various amounts of (**a**) ZIF-67 and (**b**) Pyrolyzed ZIF-67.

Thermogravimetric analysis (TGA) was done to evaluate the outcome of additive MOF on the thermal stability of the films from 25 to 600 °C at a 10 °C ramp rate per minute under a N_2_ atmosphere. The weight change was observed with the time duration as shown in Figure 5a,b. The graphs of the percentage mass loss to time show three different stages of mass loss. The first stage of mass loss is from 25 to 100 °C, where the loss is ascribed to the loss of the moisture that is loosely bound to the surface of the films [41]. The second stage mass loss was attributed to the deterioration of the composite materials e.g., decomposition of the side chain of the nanocomposite membranes. This is the greatest contributing factor to the decrease in mass along with dehydration and the formation of volatile matter at 200 to 450 °C [42]. The mass loss in this stage is around 80% of the total mass. PVA/starch exhibited lower weight loss due to the decomposition of the branched chain of the polymer, hydrogen bonds, and the remaining solvents in the pores of ZIF-67 nanoparticles. Above this range, the mass loss is higher because of the decomposition of the carbonaceous matter [43].

By adding the ZIF-67 into PVA and starch composite, weight loss decreased as the loading rate escalated, but after 450 °C, ZIF-67 started decomposing, as shown in Figure 5a,b. Further increases in temperature would decompose the carbonaceous matters of the PVA/starch and the ZIF-67 [44]. The intermolecular gap of the ZIF-67 and composite films increased in porosity after pyrolysis. It showed a decline in the thermal properties and resisted the decomposition of the products. With the incremental increase in temperature, metallic properties and crystallinity enhanced depending on the mass loss [45]. It is interesting to note that at the highest loading i.e., 10 wt.% PZIF-67, weight loss was not very substantial compared to the 4 wt.% pyrolyzed ZIF-67, which is due to aggregation of PZIF-67 particles [46]. In summary, ZIF-67 affected the thermal stability of the PVA/starch film. However, adding different amounts of ZIF-67 to the blend films improved the mechanical strength and thermal stability of the membranes.

### 4.4. Surface Area Analysis

**Figure 6:**
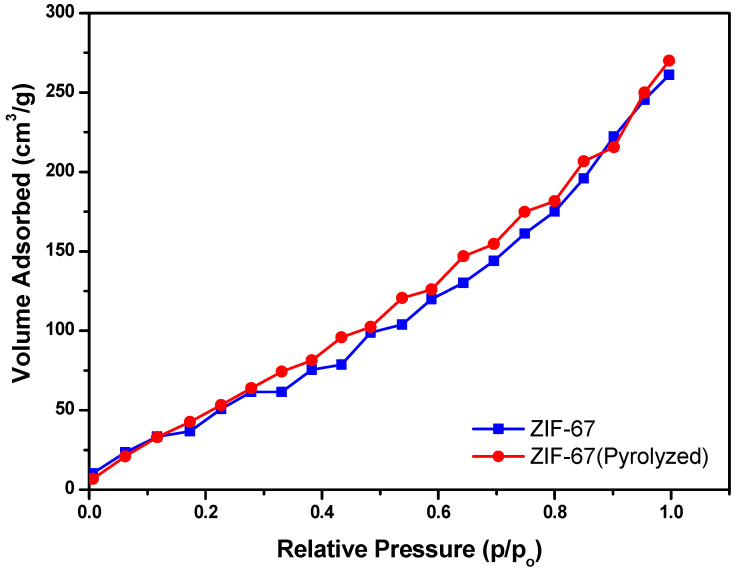
Surface area analysis by BET characterization of ZIF-67 and Pyrolyzed ZIF-67.

The structural framework of ZIF-67 and the pyrolyzed ZIF-67 was analyzed using liquid N_2_ adsorption as shown in Figure 6. International Union of Pure and Applied Chemistry (IUPAC) proposed the standard classification for the information of porous structures in terms of isothermal types I, II, and III. It can be seen from results that ZIF-67 and pyrolyzed ZIF-67 show type III isotherms [47]. The pore size, pore volume, and surface were analyzed using the isotherm. Furthermore, it is observed that the lower pressure adsorption was very low at (0.1–0.3 p/p°) in the beginning. However, as the pressure increased (0.4 to 0.99 p/p°), a significant increase in the adsorption of ZIF-67 and pyrolyzed ZIF-67 is observed.

The measured surface area of a single-point BET was 200.4 m^2^/g and 246 m^2^/g for the ZIF-67 and pyrolyzed ZIF-67, respectively. The BET results also revealed that the potential of using MOFs above 500 °C enhanced the adsorption capacity of the films due to an increase in the surface area [48]. The Langmuir surface area was also enhanced by the pyrolysis of ZIF-67 because Langmuir surface area depends on the adsorption ability of the adsorbent. After the pyrolysis, the surface area of ZIF-67 increased, yielding the microspores arrangement that is favourable for increasing the strength of the composites as shown in Table 3. It was observed that with the increase in porosity, the volume of the microspores also increased. This could enhance the thermal properties of the ZIF-67 doped PVA/starch films.

### 4.5. Mechanical Strength of ZIF-67 Doped PVA/Starch Films

**Figure 7:**
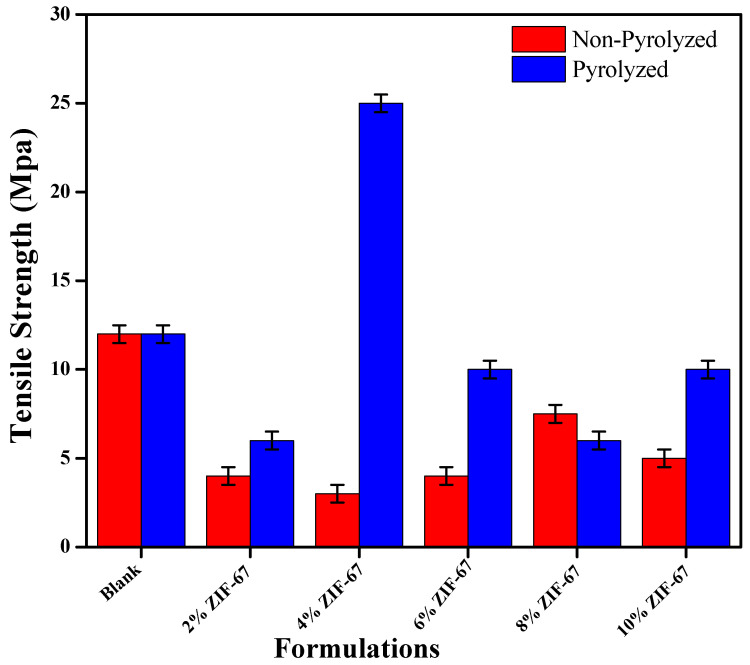
Tensile strength of PVA/starch nanocomposite films containing pyrolyzed ZIF-67 and non-pyrolyzed ZIF-67.

**Figure 8:**
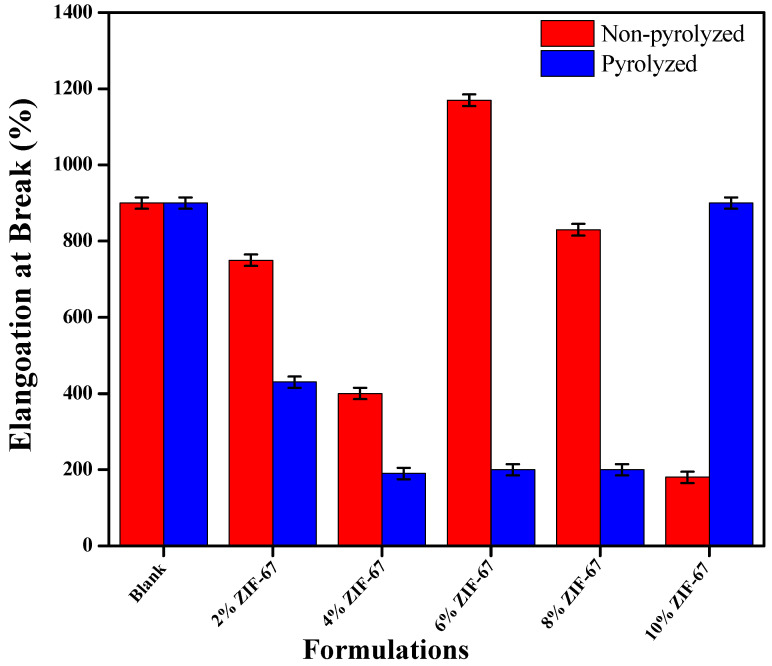
Elongation at break of PVA/starch nanocomposite films containing pyrolyzed ZIF-67 and non-pyrolyzed ZIF-67.

The mechanical properties are significant in order to analyze the strength and durability to resist extrinsic forces in application of these films as packaging materials. For their mechanical properties in packaging applications, tensile strength and strain experiments were performed to evaluate the rigidity, flexibility, strength, and elasticity of all of the synthesized formulations. The rigidity of the intermolecular framework created at the internal region is also supported by the strength and elongation at break measurements [38]. Figure 7 shows the tensile strength (MPa) and Figure 8 for engineering the elongation at break (%) plots for PVA–starch and ZIF-67 films. A slight decline in tensile strength was observed at 2 wt.% ZIF-67 due to the addition of the MOFs. By the addition of the pyrolyzed ZIF-67, the tensile strength of the PVA/starch shows incremental increases, and the highest strength value obtained at 4 wt.% was 25 MPa.

After that, the concentration of the ZIF-67 amount continued to increase the tensile strength of the film, only to decrease again, and the tensile strength was overall lower in comparison to the neat PVA film. This can be ascribed due to the sintering and highly porous structure of the ZIF-67, which enormously lowers the uniformity of the composite membrane [49]. It can be observed from the results that the percentage strain at a breakpoint is reduced as related to neat PVA/starch films. It indicates that the flexibility of the PVA/starch film is reduced because of the uniform distribution of ZIF-67. After 4 wt.%, the ZIF-67 nanoparticles continuously increased the strain rate, which incremented for the non-pyrolyzed ZIF-67 due to the agglomeration structure of the film but remained constant for the pyrolyzed composite film [50]. Comparing the maximum tensile strength endured by membranes containing ZIF-67 and pyrolyzed ZIF-67, the composites containing pyrolyzed ZIF-67 are stronger. It can be seen through the results that the strength of pyrolyzed MOFs is greater than the ZIF-67, as strain rate of ZIF-67 is greater than that of PZIF-67. This can attribute to the stronger interactions and optimum dispersion rate between the exfoliated ions and the polymer matrix. Reduction in ductility represents the formation of an inflexible PVA framework and starch chains at an intermolecular region of nanocomposites [24].

### 4.6. Chemistry Analysis of PVA/Starch with ZIF-67

**Figure 9:**
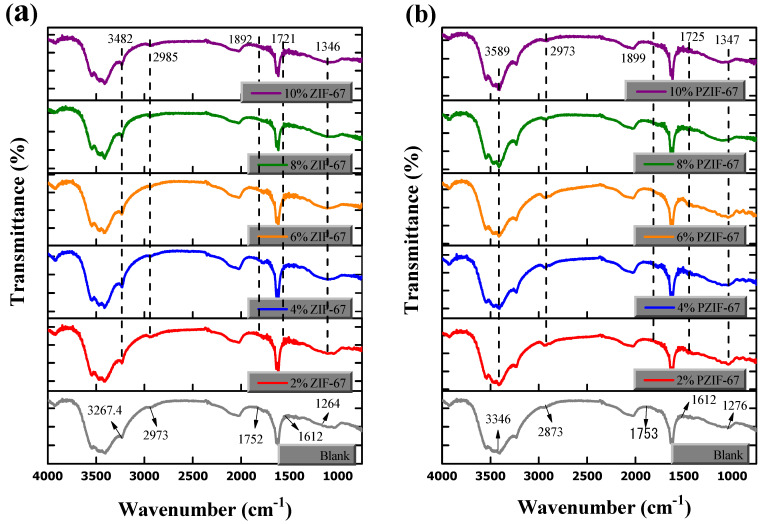
FTIR analysis PVA/starch composite films; (**a**) Containing ZIF-67 and (**b**) Various amounts of Pyrolyzed ZIF-67.

The spectrum in Figure 9a,b provides the characteristic spectrum of the ZIF-67 without pyrolysis and with pyrolysis, respectively. The absorption peaks remain the same in all of the composite films with 2 to 10 wt.% ZIF-67 loading, showing that the chemical structure of the films is not affected by the addition of the ZIF-67. The broad and bending vibration of the hydrogen bond (–OH group) of PVA and PVA/starch blends appeared at 3500–3000 cm^−1^ due to the PVA and water stretching frequency. With increments in the ZIF-67 concentration, the O–H bands shifted to a higher absolute frequency area and the related bands of the film became stronger and deeper. The various compositions of the ZIF-67 added to the PVA/starch blend showed good chemical attraction and compatibility in the chain structure due to the presence of the organic imidazole ring. All spectra demonstrate the characteristic absorption bands of pure PVA, which are at 3548, 2041, 1745, 1465, 1430, and 1275 cm^−1^ [35].

The vibrational peaks near 2973 cm^−1^ showed the C–H bond, C=C stretching, and CH_2_ groups observed in the FTIR spectra of the PVA/starch blends, indicating the successful blending of PVA with starch [51]. The infrared frequency peak closer to the wavenumber 1752 and 1246 cm^−1^ were caused by the C=O and C–C stretching, respectively. The existence of nitrogen in the ZIF-67 form the carbon and hydrogen bond in the polymer chains enhances the film formation [52]. C=N stretching is observed at 1612 cm^−1^ and shows the presence of ZIF-67, while the peak near 1064 cm^−1^ is ascribed to C–O stretching of PVA and starch presence. The C–H wagging of PVA/starch appeared at 943 cm^−1^ and shifted to 926 cm^−1^. The sharp bands have been ascribed to the group’s stretching mode of the C–C at 917 cm^−1^ [52]. As the amount of the ZIF-67 increased, the formation of a hydroxyl bond between the ZIF-67 and film is observed, which causes the hydrogen bond becomes stronger.

### 4.7. Interaction Between ZIF-67 and Polymer

**Figure 10:**
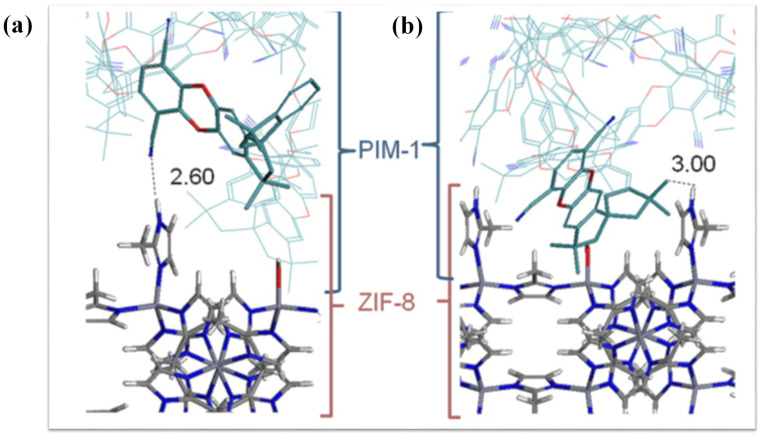
ZIF-8/PIM-1 molecular relation. (**a**) A 2.60 intermolecular interaction bond length and (**b**) a chain reaction 3.00 structural length.

This research revealed that compared to traditional polymer nanocomposites where solid nano-sized materials are used as additives, intermolecular interaction also works as a deterministic role in the establishment of the properties of MOFs-depending nanocomposites [53]. The molecular interaction between ZIF-67 and polymer chains is responsible for the thermo-mechanical behaviours of the packaging films. The relation between the MOFs ZIF-8 and polymers is shown in Figure 10. In the same way, ZIF-67 creates chain reactions with polymers and forms homogenous blends. However, wide research on the role of the intermolecular relations involved molecular packing at interphase as well as the thermomechanical characteristics of these nanocomposites films still needs improvement [24].

## 5. Conclusions

The successful synthesis of ZIF 67 was carried out using de-ionized water instead of the previously used conventional methanol technique. The addition of ZIF-67 to PVA–starch films was studied, and the concentration of MOFs was varied to analyze the change in the physical characteristics of the films, such as strength and thermal stability. The effect of ZIF-67 and pyrolyzed ZIF-67 was studied, and an improvement in mechanical strength was observed. The pyrolysis of the ZIF-67 enhances the surface area of the material. The amount of ZIF-67 in the PVA–starch blend was optimized for mechanical strength, and it was observed that the highest mechanical strength of 25 MPa was at 4 wt.% pyrolyzed MOFs. The SEM and XRD related to morphology and chemical structure, respectively, revealed that with the percentage increase of MOF in a PVA–starch film, an increase in the roughness and metal content of the blend was observed. Thermal properties were also enhanced to some extent because of the loading of nanomaterials i.e., the 4 wt.% of pyrolyzed ZIF-67 that is helpful for different packaging applications. FTIR results revealed the chemical cross-linking and strong hydrogen bonding between the PVA/starch with ZIF-67. In summary, pyrolyzed or non-pyrolyzed ZIF- loaded in PVA/starch membranes create the optimum performance and properties for packaging applications. The obtained membranes can be examined for antimicrobial applications in food packaging.

## Figures and Tables

**Table 1:** The concentration of ZIF-67 in PVA–Starch films with a corresponding amount of additive.

Amount of ZIF-67 (wt.%)	Amount of ZIF-67 (g)	Amount of PVA (g)	Amount of Starch (g)
Blank	0.00	0.94	0.56
2	0.03	0.92	0.55
4	0.06	0.90	0.54
6	0.09	0.88	0.53
8	0.12	0.86	0.52
10	0.15	0.84	0.51

**Table 2:** The concentration of cobalt in ZIF-67 and pyrolyzed ZIF-67 PVA/starch nanocomposite membranes.

Additive Concentration	Cobalt Concentration before Pyrolysis	Cobalt Concentration after Pyrolysis
(wt.%)	(wt.%)	(wt.%)
Blank	0	0
ZIF-67	22.92	16.47
2	0.11	0.01
4	0.18	0.03
6	0.22	0.05
8	0.25	0.07
10	0.26	0.10

**Table 3:** Textural parametric quantities of MOFs ZIF-67 and pyrolyzed ZIF-67.

Sample	S_BET_ (m²/g)	V_mic_ (cm^3^/g)	V_meso_ (cm^3^/g)	V_total_ (cm^3^/g)	Langmuir Surface Area (m^2^/g)
ZIF-67	200.4	0.15	0.31	0.40	346.3
Pyrolyzed ZIF-67	246	0.12	0.30	0.42	469.7
